# Interdisciplinary consensus statements on imaging of scaphoid fractures

**DOI:** 10.1007/s00330-026-12629-x

**Published:** 2026-06-11

**Authors:** Tobias Johannes Dietrich, James Teh, Rainer Schmitt, Alain Blum, Nina Hesse, Radhesh Krishna Lalam, Andoni Paul Toms, Fabio Becce, Patrick Omoumi, Maryam Shahabpour, Maciej Bień, Marco Zanetti, Robert Downey Boutin, Jan Fritz, Eva Llopis, Alberto Bazzocchi, P. Diana Afonso, Vasco V. Mascarenhas, Reto Sutter, Anne Cotten, Jean-Luc Drapé, Guillaume Bierry, Grzegorz Pracoń, Milko C. de Jonge, Danoob Dalili, Marc Mespreuve, Maciej Klich, Ladislav Nagy, Robert M. Szabo, Jörg G. Grünert, Emmanuel J. Camus, Gregory Ian Bain, Christophe L. Mathoulin, Florian Goehtz, Marc Garcia-Elias, Luc Van Overstraeten, Francisco del Piñal, Andrea Atzei, Riccardo Luchetti, Adrian Julian Chojnowski, Piotr Czarnecki, Fernando Corella, Michiro Yamamoto, Igor O. Golubev, Jörg van Schoonhoven, Luis Cerezal, Iwona Sudoł-Szopińska

**Affiliations:** 1 HOCH, Cantonal Hospital St. Gallen, Radiology and Nuclear Medicine, St. Gallen, Switzerland; 2 Faculty of Medicine, University of Zurich, Zurich, Switzerland; 3 Department of Radiology, Nuffield Orthopaedic Centre, Oxford University Hospitals NHS Trust, Oxford, UK; 4 Department of Radiology, University Hospital, LMU Munich, Munich, Germany; 5 Department of Radiology, University Hospital Würzburg, Würzburg, Germany; 6 Guilloz Imaging Department, Central Hospital, University Hospital Center of Nancy, UDL, Nancy, France; 7 Department of Radiology, Robert Jones and Agnes Hunt Orthopaedic Hospital, Oswestry, UK; 8 Norwich Medical School, University of East Anglia, Norwich, UK; 9 Department of Diagnostic and Interventional Radiology, Lausanne University Hospital and University of Lausanne, Lausanne, Switzerland; 10 Radiology, Hôpital Universitaire de Bruxelles—Bordet, Université Libre de Bruxelles, Brussels, Belgium; 11 Gamma Medical Center, Warsaw, Poland; 12 Radiology, Kantonsspital Baden, Baden, Switzerland; 13 Department of Radiology, Stanford University School of Medicine, Stanford, CA USA; 14 Department of Radiology, New York University Grossman School of Medicine, NYU Langone Health, New York, NY USA; 15 Hospital de la Ribera. IMSKE, Valencia, Spain; 16 Diagnostic and Interventional Radiology, IRCCS Istituto Ortopedico Rizzoli, Bologna, Italy; 17 Hospital CUF Tejo, Grupo CUF, Lisbon, Portugal; 18 Hospital Particular da Madeira, HPA, Madeira, Portugal; 19 Musculoskeletal Imaging Unit, Hospital da Luz, Lisbon, Portugal; 20 Imaging Department, Universidade Católica Portuguesa, Católica Medical School, Lisbon, Portugal; 21 Imaging, Joaquim Chaves Saúde, Lisbon, Portugal; 22 Radiology, Balgrist University Hospital, University of Zurich, Zurich, Switzerland; 23 Musculoskeletal Imaging Department, CIAL, Lille University, Lille, France; 24 Service de Radiologie B, Groupe Hospitalier Cochin, AP-HP.Centre, Université Paris Cité, Paris, France; 25 MSK Imaging, University Hospital, Strasbourg Cedex, France; 26 Department of Radiology, St. Antonius Hospital Utrecht, Utrecht, The Netherlands; 27 Radiology Department, Dorking Road, Epsom & St Helier University Hospitals NHS Trust, London, UK; 28 Department of Medical Imaging, University Hospital Ghent, Ghent, Belgium; 29 Specjalistyczna Praktyka Lekarska Chirurgia Ręki Maciej Klich, Warsaw, Poland; 30 Division for Hand Surgery and Surgery of Peripheral Nerves, Balgrist University Hospital, University of Zurich, Zurich, Switzerland; 31 Department of Orthopaedic Surgery, University of California Davis, Health System, Sacramento, CA USA; 32 Department of Hand and Plastic Surgery, Berit Klinik, Goldach, Switzerland; 33 Brussels Free University (ULB), Brussels, Belgium; 34 Hand Surgery Unit, IMPPACT Group, Lesquin, France; 35 Hand Surgery Unit, IMPPACT Group, Polyclinique du Val de Sambre, Maubeuge, France; 36 Department of Orthopaedic Surgery, Flinders University of South Australia and Flinders Medical Centre, Adelaide, SA Australia; 37 International Wrist Center, Clinique Bizet, Paris, France; 38 Clinic for Hand Surgery, Rhön Medical Center, Bad Neustadt/Saale, Germany; 39 Hand and Upper Extremity Surgery, Creu Blanca, Barcelona, Spain; 40 Hand and Foot Surgery Unit (HFSU) SPRL, Tournai, Belgium; 41 Department of Orthopaedics and Traumatology, Erasme University Hospital, Brussels, Belgium; 42 Instituto de Cirugía Plástica y de la Mano, Madrid, Spain; 43 Pro-Mano, Treviso, Italy and Ospedale San Camillo, Treviso, Italy; 44 Rimini Hand Surgery and Rehabilitation Center, Rimini, Italy; 45 Orthopaedics and Trauma Department, Hand and upper limb surgery, Norfolk and Norwich University NHS Trust Hospital, Norwich, UK; 46 Traumatology, Orthopaedics and Hand Surgery, Poznan University of Medical Sciences, Poznań, Poland; 47 Spain- Orthopedic and Trauma Department, Hospital Universitario Infanta Leonor, Madrid, Spain; 48 Hand Surgery Unit. Hospital Universitario Quirónsalud Madrid, Madrid, Spain; 49 Surgery Department, School of Medicine, Universidad Complutense de Madrid, Madrid, Spain; 50 Department of Human Enhancement and Hand Surgery, Nagoya University, Nagoya, Japan; 51 National Medical Research Centre of Traumatology and Orthopaedics named after NN Priorov Hand Trauma and Microsurgery Division, Moscow, Russia; 52 Radiology Department, DMC-Diagnóstico Médico Cantabria, Santander, Spain; 53 Department of Radiology, National Institute of Geriatrics, Rheumatology and Rehabilitation, Warsaw, Poland

**Keywords:** Diagnostic imaging, Surveys and questionnaires, Wrist injuries

## Abstract

**Objectives:**

To establish evidence-based consensus statements on imaging of scaphoid fractures.

**Materials and methods:**

Nineteen hand surgeons formulated a preliminary list of eleven questions on imaging of scaphoid fractures. Based on this preliminary list, radiologists crafted statements considering literature and their clinical experience, then refined them through an iterative Delphi process to revise the questions and statements. A maximum of three Delphi rounds was scheduled until group consensus was achieved for an individual statement, whichever arose first. Twenty-eight radiologists drafted the statements and acted as Delphi panellists. Panellists rated their level of agreement with each statement on an 11-point numeric rating scale, the score ‘0’ indicated complete disagreement and the score ‘10’ indicated complete agreement, respectively. Group consensus was specified as a score of ‘8’ or higher for ≥ 23/28 panellists.

**Results:**

Eight of eleven questions and statements achieved group consensus in the first Delphi round. The remaining three questions and statements achieved group consensus in the second Delphi round, indicating more controversial topics. It was agreed that radiographs are the initial imaging technique of choice for suspected scaphoid fractures. MRI or CT are advocated for suspected radiographically occult scaphoid fractures. CT is the method of choice for assessment of osseous consolidation. Contrast-enhanced MRI is the preferred imaging modality for assessing vascularisation of scaphoid nonunion. CT is the most valuable technique in the postoperative evaluation of scaphoid fractures.

**Conclusion:**

Delphi-based consensus statements suggest imaging pathways to diagnose scaphoid fractures, assess osseous fracture consolidation and evaluate pre- and postoperative fractures.

**Key Points:**

***Question*** *How can an international and interdisciplinary team of hand surgeons and musculoskeletal radiologists develop practical consensus statements on imaging of scaphoid fractures*?

***Findings*** *All eleven statements achieved group consensus among experts using the Delphi technique for consensus-building. Imaging pathways were suggested to diagnose and assess scaphoid fractures*.

***Clinical relevance statement*** *International, interdisciplinary and evidence-based consensus statements on imaging of scaphoid fractures were achieved using the Delphi technique. The focus of the statements was to diagnose scaphoid fractures, assess osseous fracture consolidation and evaluate pre- and postoperative fractures*.

**Graphical Abstract:**

**Figure:**
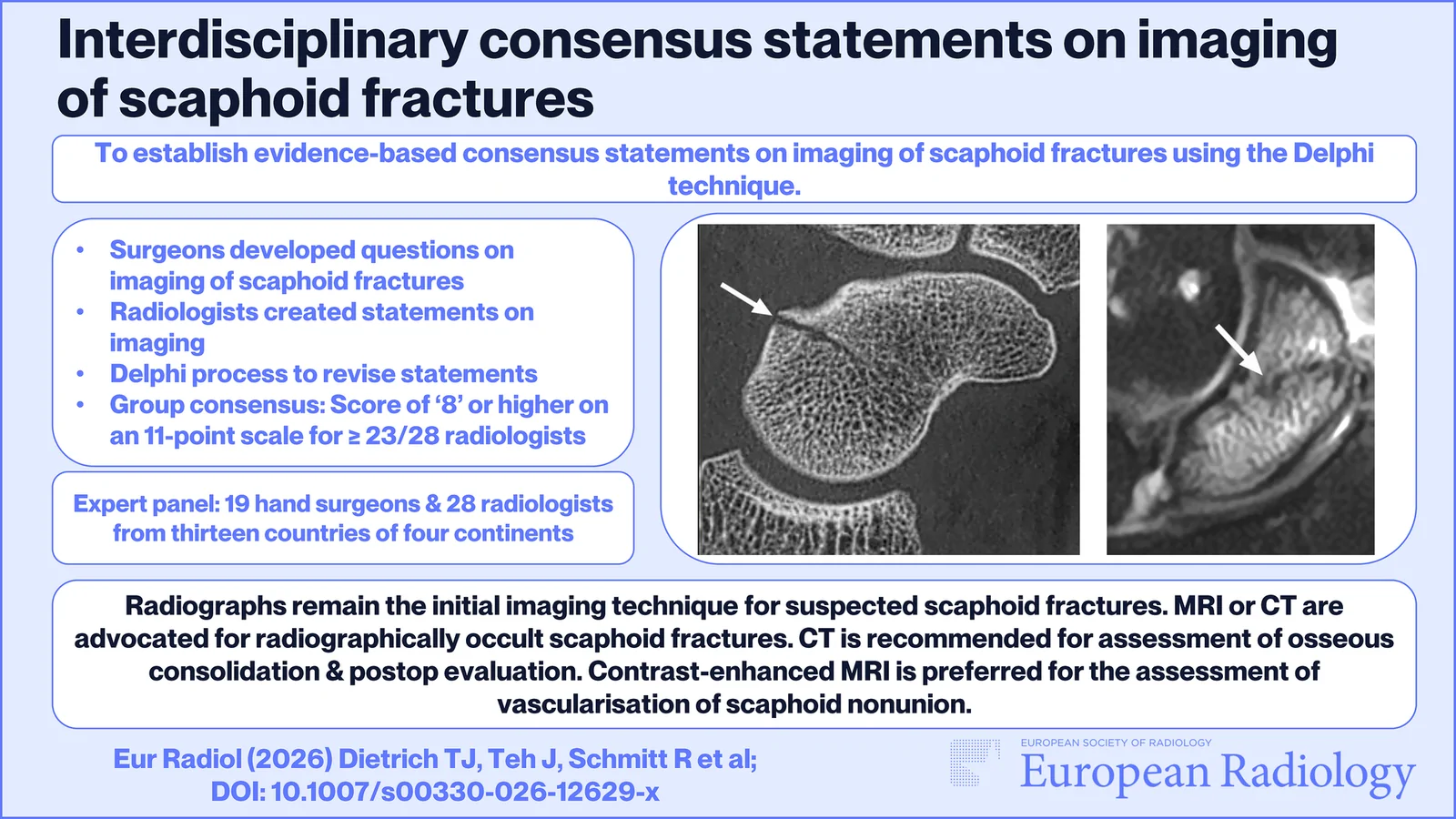

## Introduction

Scaphoid fractures are a common indication for imaging. However, the choice of the most appropriate imaging technique can be ambiguous. The diagnosis and assessment of scaphoid fractures on radiographs and cross-sectional images may remain challenging. A clinically suspected scaphoid fracture should be confirmed on imaging, but often also needs to be excluded. Radiographs are frequently the initial imaging technique due to their wide accessibility, short reading time and reasonable diagnostic efficiency [1, 2]. A rate of false negatives, also known as radiographically occult scaphoid fracture, was reported between 16% and 19%, and equivocal results are common, even among experienced readers [2, 3]. It was shown that a commercially‑available artificial intelligence algorithm significantly increases radiologists’ sensitivity (0.66–0.70) and negative predictive value (0.59–0.62, *p* < 0.0001) for diagnosing wrist and hand fractures on radiographs, without significant influence on specificity (0.89 vs 0.89) or positive predictive value (0.89 vs 0.90) [4, 5].

Relying solely on repeat radiographs within six weeks of immobilisation and activity restriction is no longer an acceptable approach for managing suspected scaphoid fractures [1, 6]. Cross-sectional imaging techniques such as CT, and magnetic resonance imaging (MRI) serve as a second step to diagnose or exclude a scaphoid fracture [1, 2, 7]. The indication for CT and MRI depends on several features, particularly diagnostic accuracy, availability, decision-making for subsequent treatment, cost-effectiveness, and radiation protection considerations. Furthermore, imaging is frequently utilised for evaluation of fracture healing and complications such as delayed union, nonunion, and malunion of scaphoid fractures, including humpback deformity. It also serves to assess scaphoid fracture instability, vascularisation in cases of suspected necrosis, and preoperative evaluation, including scaphoid nonunion advanced collapse (SNAC) and wrist osteoarthritis. Imaging is also essential for postoperative follow-up of scaphoid fractures [1, 2].

Although national guidelines are available for the diagnosis and management of scaphoid fractures, there are no specific guidelines for imaging scaphoid fractures by an international panel [1, 8–10]. An interdisciplinary team comprising hand surgeons and musculoskeletal radiologists was formed to develop consensus statements on scaphoid fracture imaging for diagnosis, preoperative and postoperative evaluation [11–13].

The purpose of this research was to establish evidence-based consensus statements on imaging of scaphoid fractures among experts using the Delphi technique.

## Materials and methods

### Panellists

The expert group of this work consisted of nineteen hand surgeons from Spain (*n* = 3), France (*n* = 2), Germany (*n* = 2), Italy (*n* = 2), Poland (*n* = 2), Switzerland (*n* = 2), Australia (*n* = 1), Belgium (*n* = 1), Japan (*n* = 1), Russia (*n* = 1), UK (*n* = 1) and USA (*n* = 1) and twenty-eight radiologists from Switzerland (*n* = 5), France (*n* = 4), UK (*n* = 4), Poland (*n* = 3), Belgium (*n* = 2), Germany (*n* = 2), Portugal (*n* = 2), Spain (*n* = 2), USA (*n* = 2), Italy (*n* = 1) and the Netherlands (*n* = 1). Twenty-seven of 28 radiologists were already committed to two previous consensus statements on imaging of wrist injuries [12, 13]. One additional radiologist (N.H.) was recommended by one panellist (R.S.) to ensure alternation of generations. All experts were either specialists in hand surgery or wrist imaging and demonstrated a high proficiency in clinical, scientific, and educational activities [12, 13]. All 28 radiologists who served as Delphi panellists were also involved in formulating the statements. Overall, four panellists’ meetings were conducted as a videoconference (October 2023), at two annual meetings of the European Society of Musculoskeletal Radiology (ESSR; 2023, 2024) and the European Congress of Radiology (ECR; 2024) on-site or as hybrid sessions combining both on-site and video-conferencing methods.

### Questions

Initially, all nineteen hand surgeons received emails with the following text: “The purpose of this step is to kindly ask you, as hand surgeons, to develop questions on imaging of scaphoid fractures to be put to radiologists. Your responses will be the basis for “Interdisciplinary consensus statements on imaging of scaphoid fractures”. Thus, you are kindly invited to provide your perspective on uncertainties and challenges in imaging of scaphoid fractures, as well as to address expectations on imaging and radiologists for decision-making of scaphoid fractures in daily clinical practice.” All questions from the hand surgeons were consolidated and structured into a preliminary list of eleven questions on imaging of scaphoid fractures (Table 1) by one hand surgeon (M.K.) and four radiologists (T.J.D., M.B., L.C., and I.S.-S.).

**Table 1:** Preliminary list of eleven questions on imaging of scaphoid fractures proposed by hand surgeons

No	Question
1	Which imaging techniques can accurately diagnose acute scaphoid fractures?
2	Which imaging protocol is suitable for the work-up of suspected radiographically occult scaphoid fracture?
3	Which imaging modality can accurately differentiate acute and chronic scaphoid fractures?
4	Which imaging modality can evaluate the instability of scaphoid fractures?
5	What terminology and descriptions of the imaging report are suitable for scaphoid fractures?
6a	Which imaging techniques can provide information of consolidation, delayed union, and nonunion?
6b	What imaging techniques allow for accurate assessment of partial or stable consolidation of a scaphoid fracture or pseudoarthrosis with partial consolidation?
7	Which imaging techniques are suitable for assessing malunion of scaphoid fractures, including the humpback deformity?
8	Which imaging modalities are suitable for the assessment of the vascularisation of the scaphoid?
9	Which imaging techniques provide the most accurate preoperative assessment for SNAC wrist?
10	Which imaging techniques can provide information of surgically treated scaphoid fractures and nonunion?

*SNAC* scaphoid nonunion advanced collapse

### Bibliography

On August 9, 2023, a literature research of the past 25 years querying ‘Web of Science’ and ‘Scopus’ was undertaken using the terms ‘scaphoid fracture’, ‘radiographs’, ‘computed tomography’, ‘magnetic resonance imaging’, ‘scintigraphy’, ‘single-photon emission computed tomography’, ‘positron emission tomography computed tomography’, ‘ultrasound’, ‘sonography’, ‘ultrasonography’, ‘sensitivity’, ‘specificity’, ‘accuracy’, ‘differentiation acute or chronic or old scaphoid fracture’, ‘instability’, ‘terminology’, ‘consolidation’, ‘pseudoarthrosis’, ‘delayed union’, ‘nonunion’, ‘malunion’, ‘humpback’, ‘deformity’, ‘vascularity’, ‘avascular necrosis’, ‘avascular’, ‘vascularization’, ‘viability’, ‘SNAC’, ‘scaphoid nonunion advanced collapse’, ‘surgery’, and ‘post-therapeutic’. Moreover, all panellists were invited to suggest articles. Sixteen meta-analyses and 165 original scientific articles were selected, continuously updated and stored in a cloud-based folder accessible to all radiologists.

### Initial statements

Task groups were established to formulate responses to each preliminary query regarding the imaging of scaphoid fractures, as proposed by the hand surgeons. All participating radiologists joined at least one task group, five radiologists (T.J.D., R.S., N.H., M.S., and L.C.) participated in two task groups. Each task group consisted of three radiologists to develop a concise statement in response to the assigned question. In addition, a brief discussion on the topic and a list of references to support the statement were requested. Each reference was assigned a scientific evidence level based on the five-point scale established by the Oxford Centre for Evidence-Based Medicine [14, 15].

### Delphi process

A maximum of three Delphi rounds was scheduled until group consensus was obtained for an individual statement, whichever arose first. Overall, two Delphi rounds were executed using survey administration software (Google Forms, https://www.google.com/forms/about/). All 28 radiologists completed the first and second Delphi surveys. Panellists rated their level of agreement with each statement on an 11-point numeric rating scale, the score ‘0’ indicated complete disagreement and the score ‘10’ indicated complete agreement, respectively.

At the panellist meetings and as part of the Delphi surveys, panellists were asked to comment on the phrasing or content of each individual question and statement on imaging of scaphoid fractures proposed by hand surgeons as listed in Table 1, specifically if their assessment did not indicate full agreement (scoring ≤ 7). Panellists’ feedback was used for iterative rephrasing of questions and statements for the upcoming Delphi round [15, 16]. Panellists were informed of the level of group agreement, including polarisation among participants, for every statement after both Delphi rounds. Feedback from panellists during meetings and as part of the Delphi surveys was blinded to other participants.

### Statistics

Group consensus was specified as a score of ‘8’ or higher for 80% or more of the radiologists (≥ 23/28 panellists). Median and interquartile range (IQR) values were determined to present polarisation among participants [12, 13, 15, 17, 18].

## Results and discussion

The literature research on diagnostic imaging of scaphoid fractures revealed a heterogeneous spread of scientific evidence between level 1 and level 5 according to the five-item scale of the Oxford Centre for Evidence-Based Medicine (Table 2) [14]. In general, a higher level of scientific evidence, e.g. values of ‘1’ and ‘2’ indicates a higher degree of evidence-based statements. In contrast, a lower-level of scientific evidence, e.g. values of ‘4’ and ‘5’ indicates a higher degree of eminence-based statements. Eight questions and statements achieved group consensus in the first Delphi round (statements #1.a, #1.b, #3, #5.a, #5.b, #6, #7 and #9). The remaining three questions and statements achieved group consensus in the second Delphi round (statements #2; #4, and #8), indicating more controversial topics. The percentages, medians and IQRs of agreement of the Delphi panel are provided in Table 2.

**Table 2:** Final questions, consensus statements and agreement among 28 panellists

No	Questions and statements	Scientific evidence level^*^	Agreement [median] (IQR)
What is the initial technique of choice to diagnose an acute scaphoid fracture?			
#1.a	Radiographs remain the initial imaging technique of choice for clinically suspected scaphoid fracture.	2	96% (27/28) [10] (10–10)
Which imaging work-up is appropriate for diagnosing radiographically occult scaphoid fracture?			
#1.b	In the event of a suspected occult scaphoid fracture, MRI or CT are advocated as an alternative to follow-up radiographs. MRI has the highest sensitivity and a high specificity for detecting scaphoid fractures. Nonetheless, CT might be chosen for its high efficiency and practical considerations. Other advanced imaging techniques are less conclusive and/or less readily available.	1	100% (28/28) [10] (9–10)
Which imaging modality is the most appropriate for differentiating acute and chronic scaphoid fractures?			
#2	If radiographs are inconclusive, CT is generally considered the best option for differentiating between acute and chronic scaphoid fractures. If uncertainty persists, MRI may be a useful further investigation. The diagnostic accuracy of radiography, CT and MRI remains uncertain due to insufficient scientific evidence.	4	89% (25/28) [10] (9–10)
Which imaging modality is the most appropriate for the evaluation of scaphoid fracture instability?			
#3	CT is the most accurate imaging modality for assessing static scaphoid fracture instability. The current scientific data lacks agreement on the most accurate imaging technique for the assessment of the dynamic instability of scaphoid fractures.	5	93% (26/28) [9] (8–10)
What information should be included in the imaging report of scaphoid fractures?			
#4	Depending on the modality used, the imaging report should consider the following information: fracture vs bone contusion, acute vs chronic fracture, the localisation of the fracture (proximal/mid/distal/tubercle), fracture orientation, displacement/dislocation, vascularity and associated wrist injuries.	4	96% (27/28) [10] (9–10)
Which imaging technique is the most appropriate for assessment of consolidation, delayed union, and nonunion?			
#5.a	While conventional radiographs remain the primary first-line modality for the assessment of scaphoid fracture consolidation, CT is the method of choice for a thorough assessment of consolidation, delayed union and nonunion.	2	100% (28/28) [10] (10–10)
Which imaging technique is the most appropriate for assessment of partial or stable consolidation of scaphoid fractures?			
#5.b	Computed tomography is the modality of choice to accurately assess osseous consolidation of a scaphoid fracture.	3	100% (28/28) [10] (10–10)
Which imaging technique is the most appropriate for assessing malunion of scaphoid fractures, including the humpback deformity?			
#6	CT is the method of choice to assess malunion of scaphoid fractures, including the humpback deformity. However, there is a lack of consensus in defining scaphoid malunion based on commonly used imaging criteria.	3	96% (27/28) [10] (9–10)
Which imaging technique is the most appropriate for the assessment of vascularisation of the scaphoid, particularly viability assessment of the proximal pole?			
#7	Contrast-enhanced MRI is the preferred imaging modality for the assessment of vascularisation of scaphoid nonunion; dynamic contrast-enhanced MRI may be suitable in some scenarios.	2	82% (23/28) [9] (8–10)
Which imaging techniques provide the most accurate preoperative assessment for SNAC wrist?			
#8	The initial assessment of the SNAC wrist should be performed with radiography. CT, MRI, CTA, or MRA can be useful to precisely determine joint damage. However, there is currently no compelling evidence to support the necessity of these modalities for preoperative planning.	3	89% (25/28) [9] (8.5–10)
Which imaging technique is the most appropriate for the evaluation of surgically treated scaphoid fractures and surgically treated nonunion?			
#9	CT is the most valuable technique in the postoperative evaluation of scaphoid fractures, especially when radiographic findings are inconclusive.	3	100% (28/28) [10] (9–10)

*CTA* computed tomography arthrography, *IQR* interquartile range, *MRA* magnetic resonance arthrography, *MRI* magnetic resonance imaging, *SNAC* scaphoid nonunion advanced collapse

^*^ Indicates scientific evidence level according to the five-item scale of the Oxford Centre for Evidence-Based Medicine [14]. In general, a higher level of scientific evidence, e.g. values of ‘1’ and ‘2’ indicates a higher degree of evidence-based statements. In contrast, a lower-level of scientific evidence, e.g. values of ‘4’ and ‘5’, indicates a higher degree of eminence-based statements

The final questions and statements are listed below:

**Question #1.a:** What is the initial technique of choice to diagnose an acute scaphoid fracture?

**Statement #1.a:** Radiographs remain the initial imaging technique of choice for clinically suspected scaphoid fracture.

Statement #1.a achieved group consensus in the first Delphi round. Ninety-six per cent (27/28) of the panellists rated the items ‘8’, ’9’ or ‘10’.

**Question #1.b:** Which imaging work-up is appropriate for diagnosing radiographically occult scaphoid fracture?

**Statement #1.b:** In the event of a suspected occult scaphoid fracture, MRI or CT are advocated as an alternative to follow-up radiographs. MRI has the highest sensitivity and a high specificity for detecting scaphoid fractures. Nonetheless, CT might be chosen for its high efficiency and practical considerations. Other advanced imaging techniques are less conclusive and/or less readily available.

Statement #1.b achieved group consensus in the first Delphi round. One hundred per cent (28/28) of the panellists rated the items ‘8’ or higher.

**Fig. 1:**
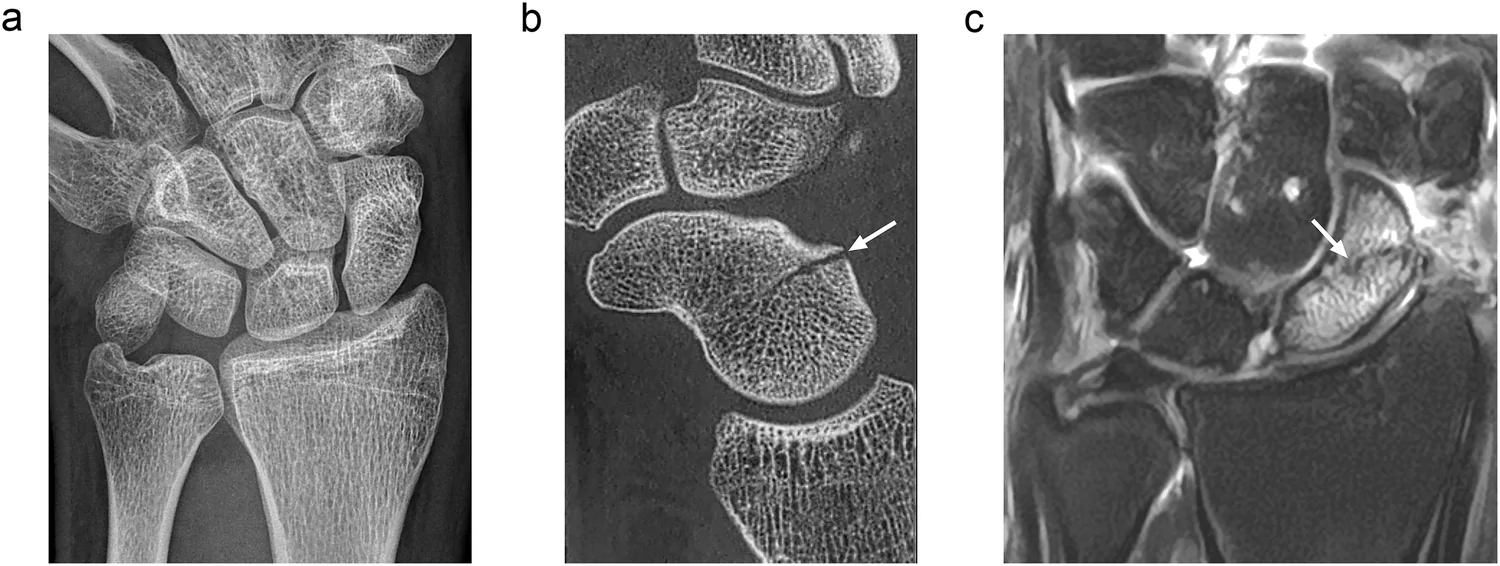
Radiographically occult scaphoid fractures in three different patients. **a** A representative view from the initial 4-view scaphoid series (posteroanterior ulnar-deviation scaphoid view) shows no definite scaphoid fracture; scaphoid nonunion was diagnosed one year later. **b** Sagittal-oblique MPR image parallel to the scaphoid’s longitudinal axis on cone beam CT, demonstrating an incomplete acute fracture on the dorsal aspect of the mid-third (arrow). **c** Coronal MR image demonstrates a hypointense fracture line (arrow) with diffuse bone marrow oedema in the scaphoid

The present consensus agreements confirm the role of radiographs as the initial technique for the diagnosis of scaphoid fractures. MRI or CT are advocated for suspected radiographically occult scaphoid fractures (Fig. 1). Opposed to the present consensus statement #1.a one may suggest: Considering the increasing availability of multidetector CT, cone-beam CT, and MRI in emergency departments, as well as the substantial rate of radiographically occult scaphoid fractures, some centres may reasonably proceed directly to cross-sectional imaging in selected patients.

Undiagnosed scaphoid fractures can potentially lead to complications such as nonunion, necrosis, carpal instability, and a characteristic pattern of progressive wrist osteoarthritis known as SNAC wrist [19, 20]. The high rate of radiographically occult scaphoid fractures, between 16% and 19% on scaphoid series, necessitates an imaging pathway to confirm or rule out wrist fractures. A Cochrane review and various meta-analyses have shown that both MRI and CT are reasonable for diagnosing radiographically occult scaphoid fractures [3, 21]. In general, scientific evidence suggests a higher diagnostic accuracy for MRI compared to CT to diagnose scaphoid fractures. A meta-analysis found that MRI provides the highest sensitivity of 94.2% and specificity of 97.7% for diagnosing scaphoid fractures, followed by CT with a sensitivity of 81.5% and specificity of 96.0% [21]. Although scientific evidence is lacking, some radiologists on the present panel are convinced that the specificity of MRI in clinical practice is lower than reported in the literature. Consequently, they assumed that MRI is related to false-positive diagnoses of scaphoid fractures due to post-contusional bone marrow oedema. Cone beam CT is a highly accurate technique for acute scaphoid fractures; a meta-analysis revealed a sensitivity of 87.7% and specificity of 99.2% to diagnose scaphoid fractures on cone beam CT images [8]. Another highly accurate technique is dual-energy CT, a study demonstrated an accuracy of 92%–100%, a sensitivity of 81%–100%, and a specificity of 95%–100% to diagnose scaphoid fractures [22]. A promising technology is photon-counting detector CT, larger study cohorts to diagnose scaphoid fractures are not available yet [23]. The scaphoid is orientated at a double-oblique angle of approximately 45 degrees to the standard orthogonal imaging planes. Thus, the localisation and course of scaphoid fractures are most accurately determined on double-oblique acquired or reconstructed cross-sectional images [1].

**Question #2**: Which imaging modality is the most appropriate for differentiating acute and chronic scaphoid fractures?

**Statement #2:** If radiographs are inconclusive, CT is generally considered the best option for differentiating between acute and chronic scaphoid fractures. If uncertainty persists, MRI may be a useful further investigation. The diagnostic accuracy of radiography, CT and MRI remains uncertain due to insufficient scientific evidence.

Statement #2 achieved group consensus in the second Delphi round. Eighty-nine per cent (25/28) of the panellists rated the items ‘8’ or higher. The second-lowest level of scientific evidence on the five-point scale by the Oxford Centre for Evidence-Based Medicine was assigned for statement #2.

Literature research did not reveal any study investigating the diagnostic accuracy of imaging to differentiate acute and chronic scaphoid fractures. The most debated issue among panellists about statement #2 was whether CT or MRI allows a more accurate differentiation between acute and chronic scaphoid fractures. The choice of imaging modality depends on the clinical context, availability, and the specific characteristics of the fracture. In some patients, a combination of radiographs, CT and MRI may be employed for a comprehensive assessment [3, 22, 24, 25].

**Question #3:** Which imaging modality is the most appropriate for the evaluation of scaphoid fracture instability?

**Statement #3:** CT is the most accurate imaging modality for assessing static scaphoid fracture instability. The current scientific data lacks considerable evidence on the most accurate imaging technique for the assessment of the dynamic instability of scaphoid fractures.

Statement #3 achieved group consensus in the first Delphi round. Ninety-three per cent (26/28) of the panellists rated the items ‘8’ or higher. The lowest level of scientific evidence on the five-point scale by the Oxford Centre for Evidence-Based Medicine was assigned for statement #3.

**Fig. 2:**
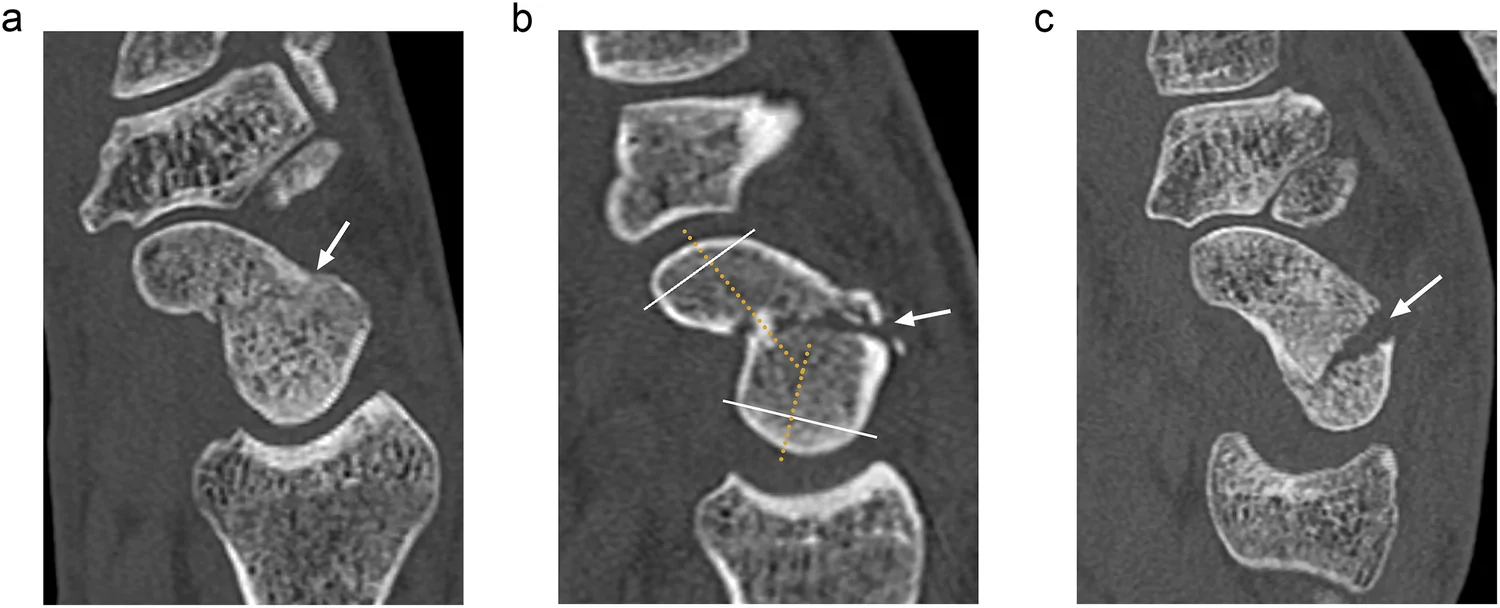
CT assessment of scaphoid fracture stability in three different patients. **a** Sagittal-oblique MPR image parallel to the scaphoid’s longitudinal axis showing a stable waist fracture (arrow) with no displacement or deformity. **b** Sagittal-oblique MPR image parallel to the scaphoid’s longitudinal axis in another patient, demonstrating an unstable mid-third fracture with displacement > 1 mm, dorsal angulation (arrow), and an increased lateral intrascaphoid angle > 35°. **c** Sagittal-oblique MPR image parallel to the scaphoid’s longitudinal axis, showing a proximal pole scaphoid fracture with significant displacement of the dorsal fracture margin (arrow)

According to Cooney et al [26], stable scaphoid fractures have less than 1 mm displacement, normal intercarpal alignment, or are located at the distal pole. Conversely, unstable fractures are characterised by over 1 mm displacement, signs of associated carpal instability, an intrascaphoid angle greater than 35 degrees (Fig. 2), a radiolunate angle over 15 degrees, and a scapholunate angle exceeding 60 degrees. Additional criteria of instability include bone loss or comminution, dorsal intercalated segmental instability, and proximal pole fractures [26, 27].

However, experts have not reached a consensus on the optimal measurement for scaphoid fracture displacement, the most effective imaging techniques, or the best method for selecting CT scan slices for consistent and repeatable results [28]. Evidence is very limited for dynamic assessment of scaphoid fracture instability on imaging, e.g. with flexion-extension tomography [29].

**Question #4:** What information should be included in the imaging report of scaphoid fractures?

**Statement #4:** Depending on the modality used, the imaging report should consider the following information: fracture vs bone contusion, acute vs chronic fracture, the localisation of the fracture (proximal/mid/distal/tubercle), fracture orientation, displacement/dislocation, vascularity and associated wrist injuries.

Statement #4 achieved group consensus in the second Delphi round. Ninety-six per cent (27/28) of the panellists rated the items ‘8’ or higher. The second-lowest level of scientific evidence on the five-point scale by the Oxford Centre for Evidence-Based Medicine was assigned for statement #4.

The imaging report should specify the imaging technique used and provide information about the localisation of the fracture. The scaphoid can be segmented into three parts along its longitudinal axis. Scaphoid fractures are commonly reported as proximal, middle and distal scaphoid fractures or as fractures of the scaphoid tubercle, respectively [1, 26]. Statement #4 was controversial among panellists. This may be partially explained because standardised and structured reporting is still an evolving field in MSK radiology, particularly compared to breast imaging [30–33].

**Question #5.a:** Which imaging technique is the most appropriate for assessment of consolidation, delayed union, and nonunion?

**Statement #5.a:** While conventional radiographs remain the primary first-line modality for the assessment of scaphoid fracture consolidation, CT is the method of choice for a thorough assessment of consolidation, delayed union and nonunion.

Statement #5.a achieved group consensus in the first Delphi round. One hundred per cent (28/28) of the panellists rated the items ‘8’ or higher.

**Question #5.b:** Which imaging technique is the most appropriate for assessment of partial or stable consolidation of scaphoid fractures?

**Statement #5.b:** CT is the modality of choice to accurately assess osseous consolidation of a scaphoid fracture.

**Statement #5.b:** achieved group consensus in the first round. One hundred per cent (28/28) of the panel rated the items ‘8’ or higher.

Preoperative and postoperative follow-up of scaphoid fractures is commonly based on serial radiographs to assess osseous consolidation, fracture alignment, the location of osteosynthesis material and complications related to the osteosynthesis material such as osteolysis [34–36]. Compared to radiographs, CT has a much higher accuracy for assessing consolidation and for the complications of conservative and surgically treated scaphoid fractures [34, 37–39].

**Fig. 3:**
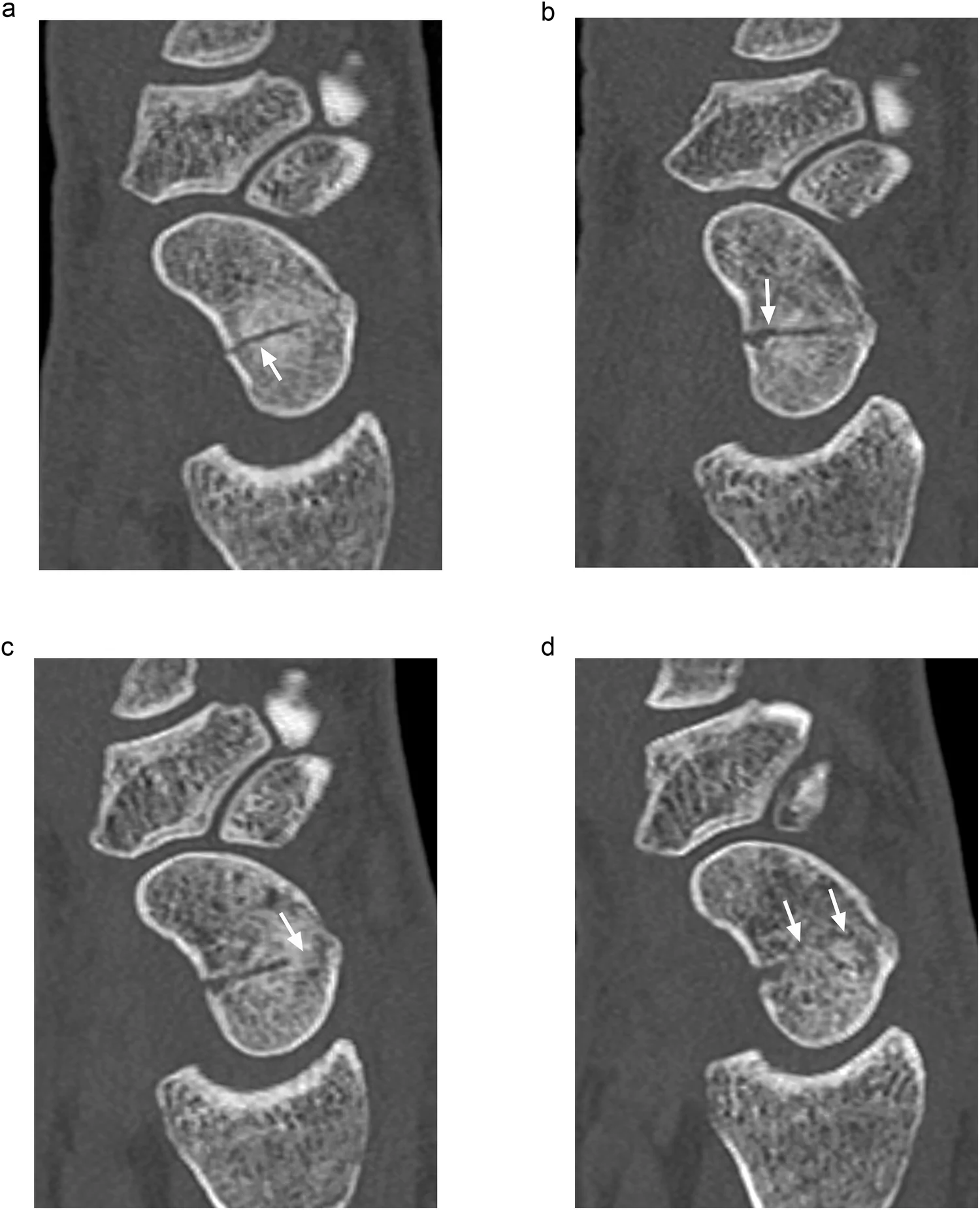
Serial CT follow-up of a stable scaphoid waist fracture healing process. Sagittal-oblique MPR images are parallel to the scaphoid’s longitudinal axis. **a** Initial diagnostic study of scaphoid waist fracture (arrow), 2 days after trauma. **b** Follow-up at 4 weeks showing slight marginal bone resorption (arrow). **c** Follow-up at 9 weeks demonstrating partial dorsal margin healing (less than 50%) (arrow). **d** Study at 16 weeks showing stable subtotal healing (greater than 50%) (arrows). The patient returned to work based on CT findings at week 16

Non-displaced scaphoid fractures are considered stable if trabecular bridging is ≥ 50% after 12 weeks (Fig. 3) [40]. However, even 30% union might be sufficient for stability [41]. A quantitative finite element analysis evaluated a cadaveric forearm with different degrees of scaphoid fracture consolidation under physiological loading. It was found that consolidation of approximately two-thirds of the fracture plane on the ulnar side, whereas approximately one-third of the fracture plane is consolidated on the radial side confirm biomechanical stability in scaphoid waist fractures that are orthogonal to the long axis of the scaphoid. Moreover, the study showed that the scaphoid fracture configuration influences the stability of partially consolidated fractures: Scaphoid fractures parallel to the radial axis require a larger consolidation area on the radial side, thus these fracture types are more prone to instability [42].

MRI is not useful for assessing the healing of scaphoid fractures, despite its efficacy in diagnosing them [43]. Acute fractures typically achieve consolidation within three months. If healing has not occurred between three and six months, the fracture is classified as a delayed union; failure to unite after six months is termed a nonunion, also known as pseudoarthrosis [34–36]. For the purposes of this work, ‘nonunion’ and ‘pseudoarthrosis’ were treated as synonymous.

**Question #6**: Which imaging technique is the most appropriate for assessing malunion of scaphoid fractures, including the humpback deformity?

**Statement #6:** CT is the method of choice to assess malunion of scaphoid fractures, including the humpback deformity. However, there is a lack of consensus in defining scaphoid malunion based on commonly used imaging criteria.

Statement #6 achieved group consensus in the first Delphi round. Ninety-six per cent (27/28) of the panel rated the items ‘8’ or higher.

A systematic review identified a lack of consensus for defining scaphoid malunion on imaging [44]. Moreover, no relationship between scaphoid malunion findings on imaging and clinical patient outcomes was found [44]. Malunion, particularly the humpback deformity, can be quantified by various measurements on imaging. However, the reliability values were relatively low, and the threshold values require further validation [45–50]. The most reliable measure for scaphoid malunion deformity was the height-to-length ratio on sagittal CT images [49, 50].

**Question #7:** Which imaging technique is the most appropriate for the assessment of vascularisation of the scaphoid, particularly viability assessment of the proximal pole?

**Statement #7:** Contrast-enhanced MRI is the preferred imaging modality for the assessment of vascularisation of scaphoid nonunion; dynamic contrast-enhanced MRI may be suitable in some scenarios.

Statement #7 achieved group consensus in the first Delphi round. Eighty-two per cent (23/28) of the panellists rated the items ‘8’ or higher.

The most controversial topic among panellists of the present consensus was the preferred imaging technique for assessing vascularisation of the scaphoid. It was agreed that contrast-enhanced MRI is the preferred imaging modality for the assessment of vascularisation of scaphoid nonunion. However, the benefit of routinely obtained dynamic contrast-enhanced MRI series for the assessment of vascularisation of the scaphoid was controversial. An evidence-based imaging work-up is preferred, but if conflicting data graded as scientific evidence level ‘2’, such as for the assessment of the viability of the proximal scaphoid fragment, are available, then controversial discussions among experts may occur [12, 13, 15, 16].

The proximal scaphoid fracture fragment is at risk of ischaemia and osteonecrosis due to its retrograde blood supply from the distal scaphoid pole. Osteonecrosis of the proximal fragment is a frequent indication for a vascularised bone graft during surgery [51, 52]. The diagnostic reference standards for assessing the viability of the proximal scaphoid fragment are the presence or absence of bleeding on acute fresh fragment surfaces and on osteotomy surfaces in scaphoid nonunion [53–59] or the histological evidence of osteoblasts in bone biopsies [53, 59–62]. Thus, accurate viability assessment of the proximal pole in scaphoid delayed union and nonunion requires surgery. In general, surgery is indicated in scaphoid nonunion to achieve joint preservation and high functional outcomes. Prolonged immobilisation up to six months may be suitable in selected patients, e.g. patients who cannot undergo surgery for any reason [63, 64].

Three MRI techniques are proposed for non-invasive evaluation of the viability of the scaphoid bone marrow: Unenhanced MRI, static and dynamic contrast-enhanced MRI. Low signal intensity on T1-weighted and T2-weighted images [53, 59, 61, 62, 65, 66] or exclusively on T1-weighted images on unenhanced MRI [54] are considered to indicate perfusion insufficiency of the proximal fragment. The reliability of unenhanced MRI is questioned for various reasons. Firstly, signal loss on T1-weighted images may be caused by fibrovascular repair tissue and new callus formation [66]. Secondly, necrosis with formation of mummified fat tissue may simulate viable bone marrow on T1-weighted images [67]. Thirdly, unrelated to the viability status, high signal on T2-weighted images is frequently observed in up to 90% of proximal scaphoid fracture fragments due to bone marrow oedema, haemorrhage, or immature callus [56].

**Fig. 4:**
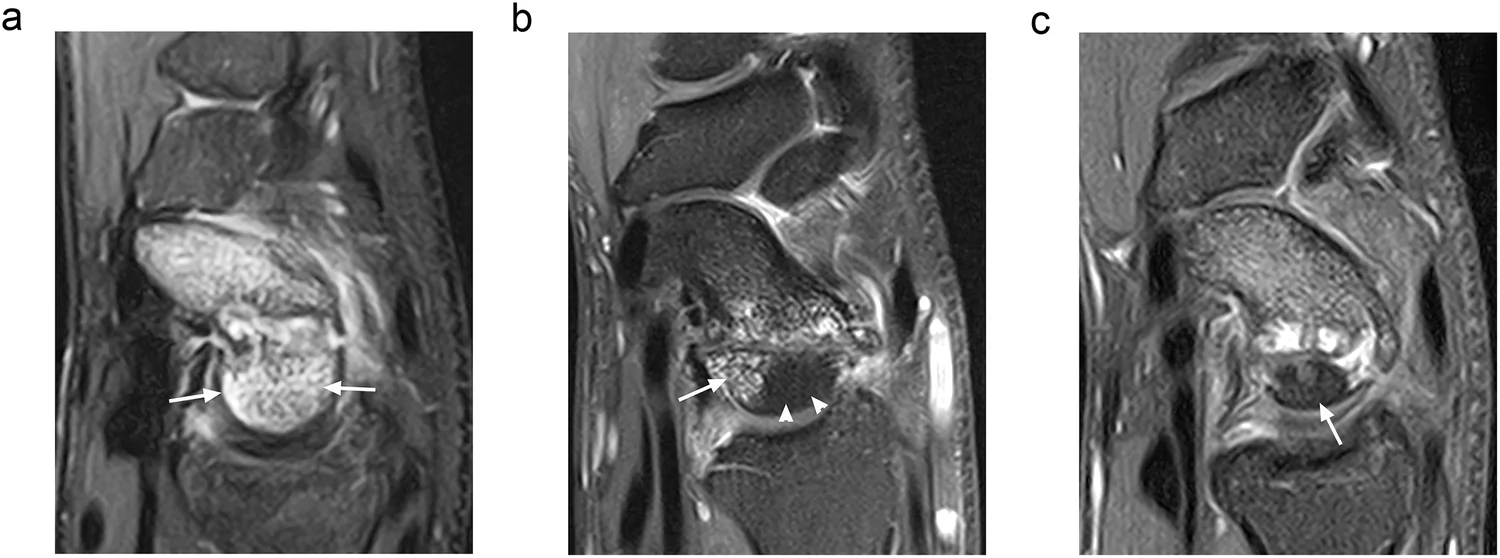
Evaluation of vascularity in the proximal scaphoid fragment on sagittal-oblique MR images along the scaphoid’s long axis after intravenous contrast administration in three different patients. **a** Intense and homogeneous contrast uptake in the proximal fragment (arrows), indicating a viable fragment, confirmed intraoperatively by punctate bleeding. **b** Limited enhancement at the palmar margin of the proximal pole (viable bone) (arrow), and absence of enhancement (necrosis) at the dorsal margin (arrowheads). The patient was treated with a vascularised bone graft from the femoral trochlea, with a good outcome. **c** Complete absence of enhancement in the proximal fragment (arrow), indicative of necrosis, confirmed intraoperatively by absence of punctate bleeding

Most authors favour contrast-enhanced MRI to assess the viability of the proximal scaphoid fragment in fresh fractures [60] and nonunions [55, 67, 68]. Contrast-enhanced MRI shows a significant correlation between the degree of enhancement and the viability of the proximal scaphoid fragment (Fig. 4). A lack of contrast enhancement on fat-suppressed T1-weighted images indicates ischaemia or osteonecrosis of the proximal fragment. Contrast enhancement shows not only viable bone marrow but also fibrovascular repair tissue around areas of nonunion or necrosis [67].

Dynamic enhanced MRI is characterised by time-resolved T1-weighted FSE sequences obtained after intravenous application of a contrast agent bolus, followed by time-intensity curves in the proximal and distal scaphoid fragments or in the radius styloid [56, 58, 60, 69]. Dynamic contrast-enhanced MRI has been suggested to provide higher diagnostic accuracy for evaluating proximal fragment viability in scaphoid delayed union and nonunion, compared to unenhanced and static contrast-enhanced MRI [58]. However, one study has indicated a lower diagnostic performance of dynamic contrast-enhanced MRI in assessing scaphoid viability when compared to standard MRI with and without contrast-enhancement in cases of scaphoid nonunion, the corresponding receiver operating characteristic curve values were 0.57 for dynamic contrast-enhanced MRI compared to values between 0.82 and 0.87 for standard MRI [56]. Thus, literature provides conflicting data for the assessment of evaluating proximal fragment viability in scaphoid delayed union and nonunion on dynamic contrast-enhanced MRI. It was suggested that a longer time interval between wrist injury and dynamic contrast-enhanced MRI may provide false-negative MRI findings for the viability of the proximal scaphoid pole [58]. The study found flattened time-related intensity curves in the proximal and distal scaphoid fragments of nonunion with a mean period of 102 ± 96 months (range: 7–156 months) between injury and dynamic contrast-enhanced MRI in false-negative MRI findings for the viability of the proximal scaphoid pole compared to determinate cases with a time interval of 17 months ± 32 months (range: 2–120 months) [58]. Although the optimal time interval between wrist injury and dynamic contrast-enhanced MRI has not been determined yet, one may suggest that in scaphoid nonunion scenarios with a time interval less than 100 months, dynamic contrast-enhanced MRI is more accurate than in patients with a longer trauma history [58].

No relationship was found between increased osteosclerosis of the proximal fragment in scaphoid nonunion on radiographs and CT images and its viability. Thus, osteosclerosis of the proximal fragment in scaphoid nonunion on radiographs and CT images is not a reliable parameter for viability of the proximal scaphoid fragment in scaphoid nonunion [53, 61, 65].

**Question #8:** Which imaging techniques provide the most accurate preoperative assessment for SNAC wrist?

**Statement #8:** The initial assessment of the SNAC wrist should be performed with radiography. CT, MRI, CTA, or MRA can be useful to precisely determine joint damage. However, there is currently no compelling evidence to support the necessity of these modalities for preoperative planning.

Statement #8 achieved group consensus in the second Delphi round. Eighty-nine per cent (25/28) of the panellists rated the items ‘8’ or higher.

**Fig. 5:**
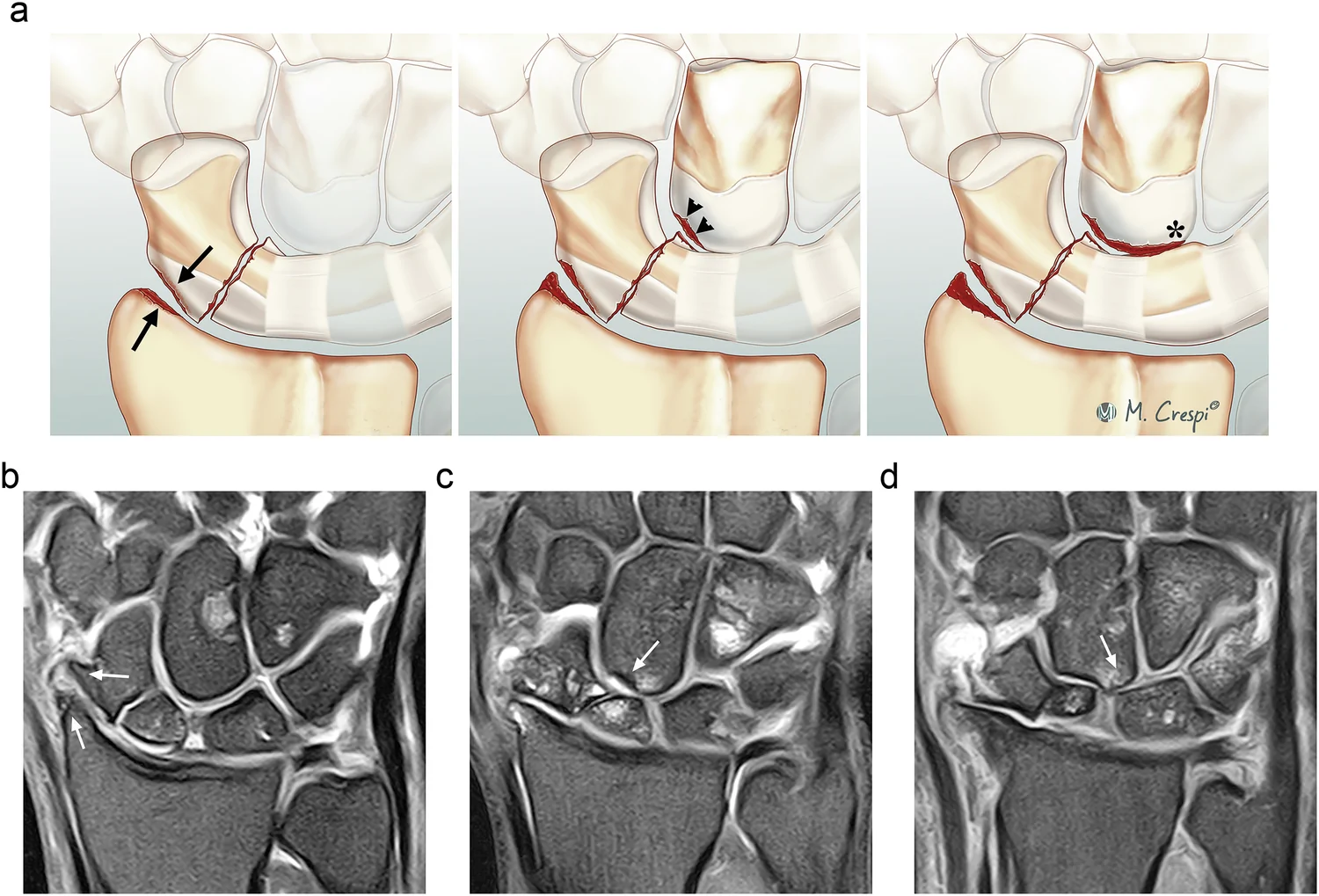
Progressive stages of SNAC wrist. **a** Schematic diagram illustrating the progressive arthritic changes in SNAC arthritis. Stages 1-3 (from left to right). Stage 1: Early degenerative changes confined to the dorsal scaphoid facet of the distal radius and the articulation with the distal scaphoid fragment (arrows). Stage 2: Involvement of the radioscaphoid joint extending to the scaphocapitate joint (arrowheads). Stage 3: Involvement of the capitolunate joint (asterisk). b-d Coronal proton density fat-suppressed MR images illustrating the progressive stages of SNAC osteoarthritis. **b** Scaphoid waist nonunion with osteoarthritis limited to the radial styloid and the opposite radial margin of the scaphoid, showing chondropathy and marginal osteophytes (stage 1 SNAC). **c** Osteoarthritis extending to the scaphocapitate joint (arrow), with focal chondropathy, slight cortical collapse, and reactive subchondral bone changes (stage 2 SNAC). **d** Advanced wrist osteoarthritis involving the radioscaphoid, scaphocapitate joints (focal cortical remodelling at the capitate margin), and involvement of the scapholunate joint (arrow) (stage 3 SNAC)

Imaging serves for the diagnosis, staging, therapeutic planning, and post-surgical follow-up of the SNAC wrist, particularly for assessment of the articular cartilage in various compartments (Fig. 5) [70, 71]. The initial imaging modality for wrist osteoarthritis are commonly radiographs [70–75]. However, CT, CT arthrography, MRI and MR arthrography (MRA) allow a more accurate cartilage assessment to evaluate the severity and distribution of the SNAC wrist [70, 71, 73, 74]. A scoping review article concluded that radiographs are consistently used in pre-operative decision-making. However, the literature lacks data on the influence of CT and MRI in selecting among various types of wrist arthrodesis and carpectomy procedures [76]. Interestingly, a decision-making analysis of hand surgeons’ preoperative planning practices for advanced carpal osteoarthritis found that most surgeons (87%) reported that radiographs are usually sufficient. In contrast, a minority of surgeons always obtain MRI or CT, with 44% thinking MRI is never useful and 38% thinking CT is never useful [77]. These inconsistencies among surgeons may partly explain why statement #8 was also a controversial topic among panellists of this work.

**Question #9:** Which imaging technique is the most appropriate for evaluation of surgically treated scaphoid fracture and surgically treated nonunion?

**Statement #9:** CT is the most valuable technique in the postoperative evaluation of scaphoid fractures, especially when radiographic findings are inconclusive.

Statement #9 achieved group consensus in the first Delphi round. One hundred per cent (28/28) of the panel rated the items ‘8’ or higher.

**Fig. 6:**
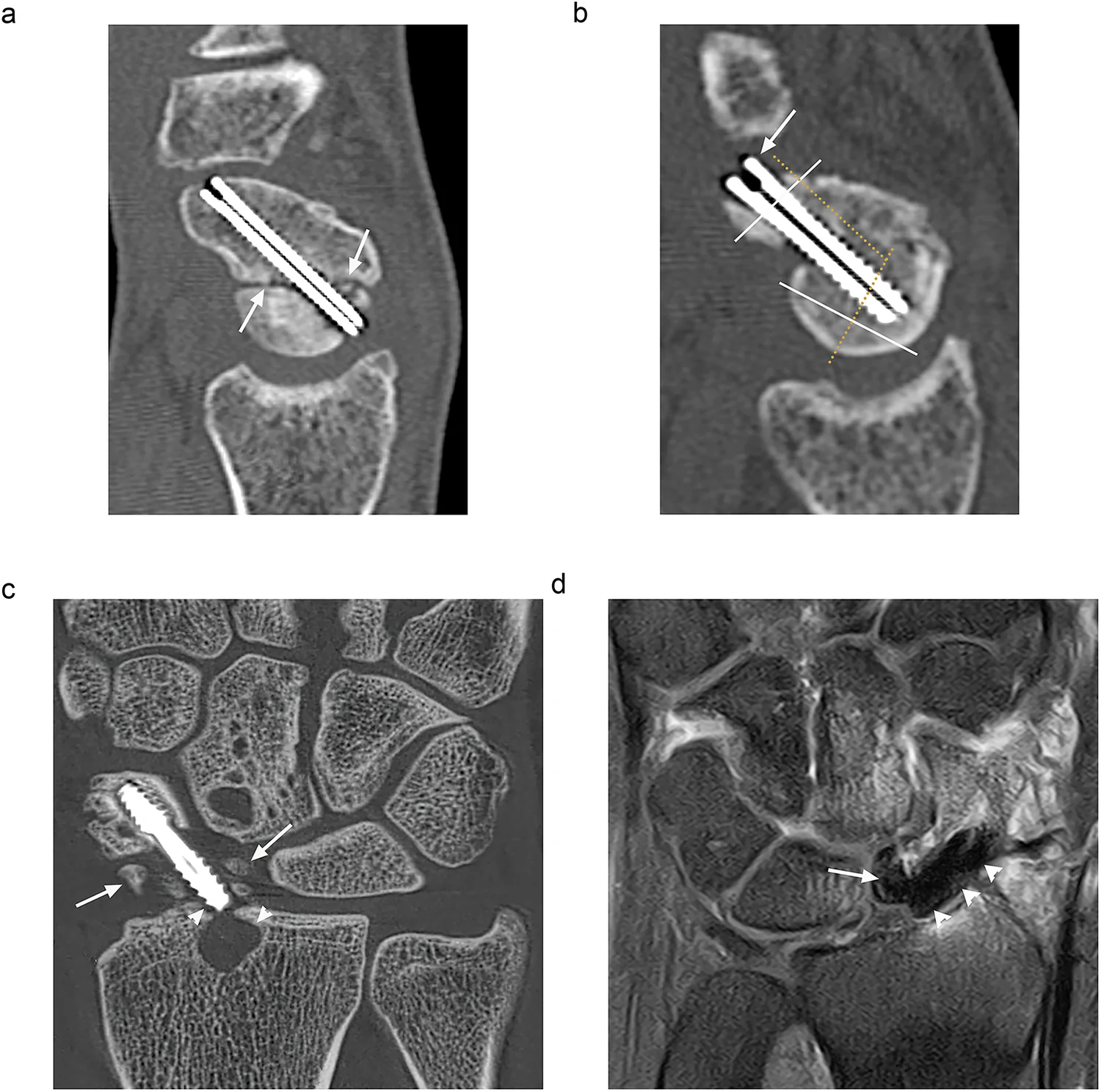
Postoperative imaging assessment of scaphoid fractures. **a** Six-month postoperative follow-up of a patient with an acute scaphoid fracture treated with an intraosseous cannulated compression screw. Sagittal-oblique MPR CT image parallel to the scaphoid’s long axis shows a complete absence of bony bridging of consolidation (arrows). **b** Six-month postsurgical follow-up of a scaphoid malunion treated with a corticocancellous bone graft and intraosseous screw. Sagittal-oblique CT image shows malunion with shortening, an increased lateral intrascaphoid angle (LISA > 35°), and protrusion of the screw head into the scaphotrapezial joint (arrow). **c** Nine-month postoperative follow-up of a scaphoid nonunion. Coronal cone beam CT shows fragmentation and collapse of the proximal pole (arrows), with screw impaction into the scaphoid fossa of the distal radius, subchondral erosion, and cyst formation (arrowheads). **d** One-year postsurgical follow-up of a scaphoid nonunion treated with a corticocancellous bone graft and intraosseous screw. Coronal fat-suppressed T1-weighted contrast-enhanced MR image demonstrates a malpositioned screw that fails to engage the proximal fragment (arrowheads) and absent enhancement of the proximal scaphoid pole, consistent with osteonecrosis (arrow)

Valuable imaging modalities for surgically managed scaphoid fractures and non‑unions accurately assess bone healing, fracture alignment, and detect implant‑related complications such as intra‑articular screw migration (Fig. 6). In routine postoperative follow‑up, plain radiographs and computed tomography (CT) are typically used [34–36, 78]. It was found that surgeons are more confident in assessing scaphoid healing after surgical treatment when they use both radiographs and CT scans, compared with relying on radiographs alone [79]. Although it was shown that contrast-enhanced MRI may diagnose osseous consolidation after treatment of scaphoid nonunions with vascularised bone grafts, MRI is generally reserved for the individual assessment of suspected complications, such as viability of bone marrow or infection [32, 33, 80].

A limitation of this work is its blend of evidence-based medicine and eminence-based medicine, even for questions and statements with a higher level of scientific evidence such as statement #1.b. Questions on imaging of scaphoid fractures were provided by experienced hand surgeons and the corresponding consensus statements were based on literature research and scientific evidence. However, for questions and statements with missing or even conflicting data, eminence-based medicine influenced the final consensus statements, particularly for the preferred assessment of vascularisation of scaphoid nonunion after intravenous contrast administration. Eight of eleven questions used the word ‘appropriate,’ however no definition for that term was provided. A bias of these consensus statements was that authors of the manuscript participated as panellists in the Delphi survey. Because the Delphi methodology relies on structured interaction, it mitigates this bias [15]. Every panellist contributes at various phases, and anonymity ensures that no single person dominates the discussion [15]. Some guidelines were added after the Delphi process was finished, so the panellists did not have access to them when they voted [8, 9]. Local variations in imaging techniques may have biased participating panellists. The present consensus statements did not address technical parameters such as appropriate CT reconstruction kernel, optimal slice thickness, essential MRI sequences and MRI field strengths.

In conclusion, the present article established evidence-based consensus statements on imaging of scaphoid fractures among experts using the Delphi technique.

## Abbreviations

CT: Computed tomography
CTA: Computed tomography arthrography
ESSR: European Society of Musculoskeletal Radiology
IQR: Interquartile range
MRA: Magnetic resonance arthrography
MRI: Magnetic resonance imaging
SNAC: Scaphoid nonunion advanced collapse
